# Factors associated with reduced substance use and treatment completion among justice-involved pregnant women

**DOI:** 10.3389/fpsyt.2026.1700103

**Published:** 2026-02-12

**Authors:** John Moore, Melissa G. Murphy, Austin Spitz, Samantha Goldfarb

**Affiliations:** 1 Florida State University College of Social Work, Tallahassee, FL, United States; 2 Temple University School of Social Work, Philadelphia, PA, United States; 3 Florida State University College of Medicine, Tallahassee, FL, United States

**Keywords:** criminal justice system, pregnant women, self-help groups, substance use, SUD, treatment, treatment outcomes

## Abstract

**Introduction:**

Pregnant women with criminal justice-system involvement (CJI) and substance use disorders (SUD) face considerable barriers that impact substance use treatment outcomes. However, limited research has investigated such factors among this population. To address this gap, we investigated associations of sociodemographic and treatment-related factors with reduced substance use and treatment completion among CJI pregnant women who utilized outpatient treatment.

**Methods:**

Data came from the 2021–2022 Treatment Episode Dataset-Discharges (TEDS-D). The sample included 1,903 CJI pregnant women discharged from outpatient treatment. Multivariable logistic regression was used to measure associations of sociodemographic and treatment-related predictors with reduced substance use and treatment completion.

**Results:**

Compared to alcohol use, marijuana use was associated with lower odds of reduced use, whereas opioid and stimulant use were each associated with lower odds of treatment completion. Self-help group attendance was associated with higher odds of both outcomes.

**Discussion:**

Findings suggest that substance type may be an important consideration for treatment planning. Examining substance-specific factors that facilitate positive treatment outcomes is a practical next step for future studies. Self-help groups were a key facilitator of positive outcomes, suggesting that the integration of peer recovery support models into formal substance use treatment services warrants consideration.

## 1 Introduction

Substance use in the perinatal period, defined as pregnancy or up to 12 months postpartum, represents a significant public health concern. In the U.S., women account for 40% of lifetime substance use disorders (SUD) and 26% of past-year SUDs (1). Data from the 2023 National Survey on Drug Use and Health indicate that approximately 5% of pregnant women in the U.S. engage in binge drinking, and 5% report past-month illicit drug use, with marijuana use being the most common (2). Perinatal substance use is linked to numerous adverse maternal, fetal, and neonatal outcomes (3). Harmful maternal and infant outcomes vary by substance use type and severity; however, obstetric complications, fetal distress, and neonatal withdrawal syndrome are well-documented (3–6). Unintended pregnancy rates are higher among women with SUD compared to women without SUD (7–9). This is significant as unintended pregnancies are linked to delayed onset of obstetric and prenatal care (10).

Many pregnant women face punitive legal consequences related to their substance use (11, 12), including heightened legal surveillance, arrest, prosecution, conviction, and child removal (13–16). Such legal consequences may exacerbate stigma, delay prenatal care, hinder treatment access, result in incarceration, and worsen maternal and fetal outcomes (13, 15, 17, 18). Importantly, pregnant women may face criminal justice-system involvement (CJI) beyond punitive criminalization laws. CJI broadly refers to those with a history of incarceration, community supervision (i.e., probation or parole), court-mandated substance use treatment, or contact with law enforcement (19). While the true prevalence is unknown, it is estimated that a sizable proportion of CJI pregnant women meet criteria for SUD (20). In this study, CJI refers to pregnant women referred to outpatient substance use treatment directly from criminal justice system institutions.

CJI treatment referral sources include pre-booking diversion programs (e.g., law enforcement referring to treatment instead of arrest), pre-sentencing diversion approaches (e.g., drug courts), mandated treatment as a condition of probation or parole, and referral following release from jail or prison. The specific type of CJI presents unique challenges that may influence treatment access and retention. Jail and prison reentry is associated with disruptions in formal and informal supports which may impede recovery (21, 22). Those under community corrections supervision or drug court surveillance are subject to complying with supervision requirements (e.g., abstinence), or else risk incarceration (23–25). Connecting CJI pregnant women with substance use issues to treatment is essential to ensuring receipt of appropriate care (20, 26, 27). Positive prenatal treatment outcomes, such as treatment completion and reduced use, mitigate the risk of maternal/perinatal harms associated with substance use. Thus, examining sociodemographic and treatment-related factors that relate with positive treatment outcomes may inform targeted interventions and treatment services.

Research in broader substance use treatment populations has identified numerous sociodemographic and treatment-related factors associated with treatment completion and reduced substance use. Differences in treatment outcomes have been found across racial and ethnic groups (28), whereas findings are mixed in terms of age (29, 30). Socioeconomic indicators, such as being employed and higher educational attainment, are generally associated with positive treatment outcomes and recovery (28, 31, 32). Moreover, stable housing and recovery-oriented living arrangements are key to promoting substance use recovery and minimizing treatment engagement barriers (33–37). Substantial variation in substance-use criminalization policies (11), as well as treatment capacity and availability (38), exists across U.S. states. Perceptions of substance use harm vary by the type of substance (2), as do maternal and fetal health risks (39–42). Furthermore, treatment outcomes differ based on the type of substance use (28, 43). Early substance use initiation (44, 45), mental disorder comorbidity (46, 47), and prior treatment history (48) are indicators of SUD severity that are integral to account for in treatment planning. Substance use self-help groups provide access to critical social supports for persons in recovery (49, 50), and self-help group participation is linked with treatment completion and reduced substance use (51–53). Longer stays in treatment are associated with positive treatment outcomes (54, 55), and persons in treatment may benefit from receiving care in intensive treatment facilities that offer more comprehensive care (56).

However, research examining sociodemographic and treatment-related factors of reduced substance use and treatment outcomes among CJI pregnant women is lacking. Research conducted among pregnant women in substance use treatment found that those with CJI were more likely to complete treatment (30, 57), potentially due to mandatory treatment participation requirements. Given the unique barriers and punitive elements of CJI that may impact treatment experiences and behavior change, the limited research on treatment outcomes among this population is a key gap in the literature. To address this, the current study examined the following research question: What sociodemographic and treatment-related factors are associated with reduced substance use and treatment completion among CJI pregnant women discharged from outpatient treatment? We focused on outpatient treatment settings given the heightened risk for substance use engagement compared to inpatient settings. This study aims to inform the development and tailoring of interventions and treatment services for CJI pregnant women by identifying treatment-related factors most strongly associated with positive treatment outcomes. Additionally, this study aimed to identify demographic subpopulations at an increased risk for adverse treatment outcomes.

## 2 Materials and methods

### 2.1 Data and sample

All study data came from the 2021–2022 Treatment Episode Dataset-Discharges (TEDS-D). TEDS-D includes annual data on adolescents and adults who are discharged from publicly funded and state-licensed substance use treatment facilities in the U.S. Treatment providers collect data and submit it to individual states, which then standardize and report the data to the Substance Abuse and Mental Health Services Administration (58). Since more than one person may receive treatment services annually, the unit of analysis for TEDS-D is a discharge from substance use treatment. TEDS-D contains information on whether a discharge received services in inpatient hospitalization, residential, or ambulatory settings (i.e., outpatient treatment) and whether medication-assisted opioid therapy was part of the treatment plan. Data files from the years 2021 and 2022 were merged into one dataset for the analysis.

TEDS-D includes variables that capture the type of treatment referral and the treatment setting from which a person was discharged. The analytic sample for this study consisted of CJI pregnant women who were discharged from outpatient substance use treatment. Pregnancy status is determined in TEDS-D by a variable that reports if a women was pregnant at the time of treatment admission. CJI was defined as referral to treatment from a criminal justice system provider. Outpatient treatment includes discharges from both non-intensive and intensive ambulatory settings. Women who were not pregnant, received services from inpatient or detox treatment providers, or were not referred to treatment by the criminal justice system were excluded. The analytic sample consisted of 1,903 CJI pregnant women discharged from outpatient substance use treatment. To examine reduced substance use, analyses were also conducted among a subsample of 1,285 discharges who attended treatment for at least thirty days.

### 2.2 Measures

#### 2.2.1 Outcomes

Study outcomes included reduced substance use and treatment completion. Reduced substance use was operationalized as a decrease in the frequency of primary substance use from treatment admission to treatment discharge. Primary substance use was defined based on a TEDS-D item that reports a person’s primary substance use at admission and discharge. TEDS-D also captures a person’s primary substance frequency at both admission and discharge. These variables contain the following response items: “1-No use in the past month”, “2- Some use”, “3-Daily use” and “-9- Missing/unknown/not collected/invalid”. A dichotomous variable was computed based on primary substance use frequency at admission and discharge. This variable indicated if a discharge either 1) reduced their substance use from admission to discharge or maintained abstinence from admission to discharge (e.g., “Daily use” to “Some use” or “No use”; or “Daily use” to “Some use”), or 2) did not reduce their use from admission to discharge or maintained the same use frequency (e.g., “Daily use” reported at both admission and discharge; or “Some use” to “Daily Use”). For the computed outcome variable, discharges who reduced their use or maintained abstinence were coded as “1-Yes” whereas discharges who maintained or increased their use were coded as “0-No”.

Treatment completion was defined as completing outpatient substance use treatment. TEDS-D contains an item reporting the reason for discharge, which includes treatment completion, dropped out of treatment, terminated by facility, transferred to another treatment program or facility, incarceration, death, and other reasons. Based on responses to this item, a dichotomous variable was created to indicate whether a respondent completed treatment or not. Completed treatment is defined in the source variable as “All parts of the treatment plan or program were completed.” Discharges who completed treatment were coded as “1-Yes” whereas those with all other discharge reasons were coded as “0-No” in the computed outcome variable.

#### 2.2.2 Sociodemographic characteristics

Sociodemographic variables assessed included age (18-24, 25-34, 35+), race/ethnicity (Black, Hispanic, Other race/ethnicity, White), educational attainment (< high school, high school, attended/graduated college), employment status (employed, not in work force, unemployed), living situation (unhoused, lived in a supervised setting, lived independently), and U.S. region (Northeast, Midwest, South, West). Insurance was considered for inclusion as a sociodemographic variable but was excluded due to high missingness (44%).

#### 2.2.3 Treatment-related factors

Treatment-related covariates included the type of primary substance use, length of treatment stay, adolescent substance use initiation, prior treatment history, mental disorder comorbidity, treatment setting type, and self-help group attendance during treatment. A variable was created that categorized the type of primary substance use as alcohol, cannabis, opioid (heroin or synthetic opioids), stimulant (cocaine, amphetamine, methamphetamine, or other CNS stimulants), or other substances. Length of treatment stay was coded as: <30 days, 31–90 days, and 91+ days. For analyses examining reduced substance use, length of treatment stay was coded dichotomously as 30–90 days or 91+ days. A dichotomous variable reported whether a discharge had a prior history of substance use treatment (yes/no). Additionally, treatment setting type was captured by a recoded dichotomous variable that demonstrated whether services were received at an intensive or non-intensive outpatient treatment setting. Mental disorder comorbidity was assessed with a dichotomous TEDS-D item that indicated the presence or absence of co-occurring mental disorder(s). TEDS-D contains information on the frequency of substance use specific self-help group attendance in the 30 days before treatment discharge (no attendance, 1–3 times, 4–7 times, 8–30 times, some attendance with frequency unknown). From this item, a dichotomous variable was created that demonstrated whether a discharge attended any self-help group in the month prior to discharge. Both reduced substance use and treatment completion were employed as covariates in analyses in which they were not the outcome.

### 2.3 Analytic plan

All data analyses were conducted using Stata 18. Missing data in the overall sample meeting inclusion criteria were low for most variables (<5%) but were higher for self-help group attendance (17%), psychiatric comorbidity (17%), and reduced substance use at discharge (20%). To account for this, we employed complete-case analysis in which univariate, bivariate, and multivariable analyses were restricted to observations with non-missing data across all model variables. Descriptive statistics presented include univariate percentages for all sociodemographic and treatment-related covariates, reduced substance use, and treatment completion. Chi-square (χ^2^) tests of independence were performed to measure bivariate associations between sociodemographic characteristics and treatment-related characteristics with reduced substance use and treatment completion.

Multivariable logistic regression was used to measure associations of sociodemographic and treatment-related factors with reduced substance use and treatment completion. Since the substance use frequency variable measures past-month usage, bivariate and multivariable analyses for reduced substance use were conducted among a subsample of pregnant women who attended treatment for at least 30 days. This was applied due to the operationalization of substance use frequency at admission and discharge, which assesses substance use within the past-month at both treatment admission and discharge. Restricting analyses to those who stayed in treatment for at least 30 days mitigates the risk of miss-labeling pre-admission substance use frequency as taking place during treatment. Statistical significance was set at α <.05, and results from the multivariable logistic regression modeling are presented as adjusted odds ratios (AOR) and 95% confidence intervals (95% CI).

## 3 Results

### 3.1 Descriptive statistics

**Table 1** shows that over 73% of discharges in the analytic sample were 25 years old or older, and 70% were White. Nearly 80% of discharges did not attend college and 74% of discharges were either unemployed or out of the workforce. Most discharges lived independently (75%). Stimulants (35%) were the most prevalent primary substance responsible for treatment, followed by opioids (21%), cannabis (19%), alcohol (18%), and other substances (6%). A plurality of discharges attended treatment for at least 91 days (48%), and most received services in non-intensive outpatient facilities (82%). Less than half of discharges initiated their primary substance in adolescence (44%), and 55% attended treatment previously. Additionally, 64% of discharges had a comorbid mental disorder. Nearly one-third of discharges successfully completed treatment (32%), and 71% reduced their substance use or maintained abstinence from treatment admission to discharge.

**Table 1:** Descriptive statistics of CJI pregnant women discharged from outpatient treatment (N = 1,903).

Characteristics	n (%)
Age	
18-24	509 (26.8)
25-34	1,115 (58.6)
35+	279 (14.7)
Race/Ethnicity	
White NH	1,330 (69.9)
Black NH	205 (10.8)
Hispanic	233 (12.2)
Other	135 (7.1)
Education	
<HS	616 (32.4)
HS	897 (47.1)
Some college/College	390 (20.5)
Employment	
Employed	502 (26.4)
Not in Labor Force	455 (23.9)
Unemployed	946 (49.7)
Living Situation	
Unhoused	99 (5.2)
Supervised	382 (20.1)
Independent	1,422 (74.7)
Region	
Northeast	408 (21.4)
Midwest	529 (27.8)
South	567 (29.8)
West	399 (21.0)
Primary Substance	
Alcohol	346 (18.2)
Cannabis	364 (19.1)
Opioids	403 (21.2)
Stimulants	668 (35.1)
Other	122 (6.4)
Length of stay in Treatment	
<=30 days	618 (32.5)
31–90 days	364 (19.1)
91+ days	921 (48.4)
Prior Treatment	
No	850 (44.7)
Yes	1,053 (55.3)
Adolescent Initiation	
No	1,063 (55.9)
Yes	840 (44.1)
Treatment Setting	
Non-intensive	1,560 (82.0)
Intensive	343 (18.0)
Any Self-Help Group Attendance	
No	1,261 (66.3)
Yes	642 (33.7)
Psychiatric Comorbidity	
No	681 (35.8)
Yes	1,222 (64.2)
Treatment Complete	
No (Includes those referred to other providers)	1,286 (67.6)
Yes	617 (32.4)
Reduced Primary Substance Use	
No	562 (29.5)
Yes	1,341 (70.5)

### 3.2 Bivariate results

Results presented in **Table 2** show that reduced substance use did not significantly differ across sociodemographic characteristics. Among treatment-related covariates, the type of primary substance use was significantly associated with reduced substance use (p=.002), with rates ranging from 72% (other substance use) to 85% (alcohol). Reduced substance use also significantly differed by length of treatment stay (p<.001), with 73% of discharges who stayed in treatment between 31–90 days reporting reduced use compared to 82% of discharges who stayed in treatment greater than 90 days. Rates of reduced substance use were also significantly higher (p<.001) for discharges who attended a substance use-specific self-help group (88%) than discharges who did not (77%). Discharges who completed treatment were also more likely (p<.001) to reduce their substance use than discharges who did not (91% vs. 71%).

**Table 2:** Bivariate associations of sociodemographic and treatment-related characteristics with treatment outcomes among CJI pregnant women discharged from outpatient treatment (%).

Characteristics	Reduced substance use	p	Completed treatment	p
Age		.39		.792
18-24	81.7		33.2	
25-34	79.4		32.5	
35+	76.7		30.8	
Race/Ethnicity		.057		<.001
White NH	78.3		27.6	
Black NH	76.5		42.9	
Hispanic	84.0		49.8	
Other	86.7		34.1	
Education		.776		.004
<HS	80.3		27.3	
HS	78.8		34.8	
Some college/College	80.5		35.1	
Employment		.415		<.001
Employed	79.8		41.8	
Not in Labor Force	81.9		35.6	
Unemployed	78.2		25.9	
Living Situation		.628		<.001
Unhoused	75.3		34.3	
Supervised	80.0		19.9	
Independent	79.9		35.7	
Region		.107		<.001
Northeast	75.0		37.5	
Midwest	81.0		33.3	
South	81.4		16.8	
West	81.6		48.4	
Primary Substance		.002		<.001
Alcohol	84.9		51.7	
Cannabis	72.7		40.1	
Opioids	79.9		25.3	
Stimulants	81.8		25.3	
Other	71.9		17.2	
Length of stay recode*		<.001		<.001
<30 days			28.8	
31–90 days	73.1		37.0	
91+ days	82.2		32.4	
Adolescent Initiation		.229		<.001
No	81.0		28.8	
Yes	78.3		37.0	
Prior Treatment		.737		.664
No	80.1		32.9	
Yes	79.3		32.0	
Treatment Type		.511		.028
Non-intensive	80.0		33.6	
Intensive	78.1		27.4	
Psychiatric Comorbidity		.198		<.001
No	81.2		47.4	
Yes	78.3		24.1	
Self Help Attendance		<.001		.616
No	76.7		32.0	
Yes	87.6		33.2	
Treatment Complete		<.001		
No	70.7			
Yes	90.6			
Reduced Primary Substance Use				
No			11.0	<.001
Yes			41.4	

*Reduced substance use analyses were restricted to discharges who remained in treatment for ≥30 days; therefore, estimates for <30 days length of stay are not reported.

Statistically significant differences in treatment completion were found across the sociodemographic characteristics of race/ethnicity (p<.001), education (p=.004), employment (p<.001), living situation (p<.001), and region (p<.001). Treatment completion rates were highest among discharges who were racial and ethnic minorities, attended or graduated college, were employed, lived independently, and lived in the West region of the U.S. Treatment completion also significantly differed by the type of primary substance use (p<.001), length of treatment stay (p<.001), adolescent substance use initiation (p<.001), mental illness comorbidity (p<.001), treatment setting (p=.028) and reduced substance use (p<.001). Compared to other within-group categories, treatment completion rates were highest among discharges who attended treatment between 31–90 days, reported alcohol as the primary substance, initiated primary substance use in adolescence, attended non-intensive treatment, did not have mental illness comorbidity, and reported reduced substance use.

### 3.3 Multivariable logistic regression modeling results

Model results presented in **Table 3** show that compared to discharges aged 18-24, discharges aged 25-34 (AOR = 0.68, 95% CI = 0.47-0.99) and discharges aged 35+ (AOR = 0.58, 95% CI = 0.35-0.95) had lower odds of reduced substance use. Discharges who were Hispanic (AOR = 1.66, 95% CI = 1.04-2.64) and discharges of Other race/ethnicity (AOR = 2.01, 95% CI = 1.08-3.73) had higher odds of reduced substance use relative to discharges who were White. Discharges who resided in the Midwest had higher odds of reduced substance use compared to discharges in the Northeast (AOR = 1.54, 95% CI = 1.03-2.30). In terms of treatment relevant factors, primary marijuana use was associated with lower odds of reduced substance use compared to primary alcohol use (AOR = 0.48, 95% CI = 0.30-0.75). Discharges who attended treatment for at least 91 days had higher odds of reduced substance use than discharges who attended treatment less than 91 days (AOR = 1.38, 95% CI = 1.02-1.88). Attending a substance-specific self-help group was associated with higher odds of reduced substance use than not attending (AOR = 1.62, 95% CI = 1.10-2.40). Completing treatment was associated with higher odds of reduced substance use compared to not completing treatment (AOR = 3.87, 95% CI = 2.73-5.48).

**Table 3:** Results from multivariable logistic regression models: Associations of reduced substance use (Model 1) and treatment completion (Model 2) among CJI pregnant women discharged from outpatient treatment.

Characteristics	Model 1 reduced substance use N=1,285	Model 2 treatment completion N=1,903
	AOR (95% CI)	AOR (95% CI)
Age (18-24)		
25-34	**0.68 (0.47-0.99)**	1.11 (0.83-1.47)
35+	**0.58 (0.35-0.95)**	1.18 (0.78-1.77)
Race/Ethnicity (White)		
Black	1.00 (0.64-1.56)	1.09 (0.74-1.58)
Hispanic	**1.66 (1.04-2.64)**	1.19 (0.83-1.71)
Other	**2.01 (1.08-3.73)**	0.69 (0.44-1.09)
Education (< HS)		
HS	0.86 (0.61-1.22)	1.19 (0.91-1.56)
Some college/College	0.99 (0.64-1.52)	1.20 (0.85-1.67)
Employment (Employed)		
Not in Labor Force	1.35 (0.89-2.06)	0.86 (0.62-1.19)
Unemployed	1.08 (0.76-1.55)	**0.73 (0.55-0.96)**
Living Situation (Unhoused)		
Supervised	1.14 (0.58-2.23)	0.70 (0.39-1.25)
Independent	1.19 (0.67-2.11)	0.97 (0.58-1.61)
Region (Northeast)		
Midwest	**1.54 (1.03-2.30)**	**0.71 (0.51-0.99)**
South	1.35 (0.81-2.24)	0.78 (0.52-1.16)
West	1.04 (0.67-2.11)	**1.49 (1.04-2.13)**
Primary Substance (Alcohol)		
Marijuana	**0.48 (0.30-0.75)**	0.97 (0.68-1.38)
Opioids	0.90 (0.54-1.50)	**0.42 (0.28-0.62)**
Stimulants	0.85 (0.53-1.36)	**0.51 (0.36-0.73)**
Other	0.59 (0.29-1.17)	**0.41 (0.22-0.76)**
Length of stay (<30 days)		
31–90 days		**4.41 (2.87-6.80)**
91+ days		**9.59 (6.51-14.12)**
Length of stay recode (30–90 days)		
91+ days	**1.38 (1.02-1.88)**	
Comorbid Mental Illness (No)		
Yes	1.07 (0.79-1.44)	**0.55 (0.44-0.70)**
Adolescent Substance Initiation (No)		
Yes	0.89 (0.63-1.24)	0.89 (0.69-1.16)
Prior Treatment (No)		
Yes	1.08 (0.78-1.48)	0.85 (0.66-1.09)
Treatment Type (Non-intensive)		
Intensive	0.85 (0.58-1.25)	0.80 (0.58-1.09)
Self Help Attendance (No)		
Yes	**1.62 (1.10-2.40)**	**2.21 (1.67-2.89)**
Reduced Primary Substance Use (No)		
Yes		**3.90 (2.84-5.38)**
Treatment Completed (No)		
Yes	**3.87 (2.73-5.48)**	

Bold face indicates statistical significance.

Regarding treatment completion, only employment and region were significantly associated with treatment completion among sociodemographic characteristics. Discharges who were unemployed (AOR = 0.73, 95% CI = 0.55-0.96) had lower odds of treatment completion compared to discharges who were employed. Compared to discharges in the Northeast, those in the Midwest (AOR = 0.71, 95% CI = 0.51-0.99) had lower odds of treatment completion while those in the West had higher odds of treatment completion (AOR = 1.49, 95% CI = 1.04-2.13). For treatment-related factors, relative to primary alcohol use, primary opioid use (AOR = 0.42, 95% CI = 0.28-0.62), primary stimulant use (AOR = 0.51, 95% CI = 0.36-0.73), and primary other substance use (AOR = 0.41, 95% CI = 0.22-0.76) were associated with lower odds of treatment completion. Length of treatment stay from 31–90 days (AOR = 4.41, 95% CI = 2.87-6.80) and 91+ days (AOR = 9.59, 95% CI = 6.51-14.12) were associated with higher odds of treatment completion relative to treatment duration of less than 30 days. Mental illness comorbidity was associated with lower odds of treatment completion compared to no mental illness comorbidity (AOR = 0.55, 95% CI = 0.44-0.70). Discharges who attended a self-help group had higher odds of treatment completion compared to discharges who did not attend a self-help group (AOR = 2.21, 95% CI = 1.67-2.89). Discharges who reduced their substance use had higher odds of treatment completion compared to discharges who did not reduce their substance use (AOR = 3.90, 95% CI = 2.84-5.38).

## 4 Discussion

The present study examined sociodemographic and treatment-related predictors of reduced substance use from treatment admission to treatment discharge and treatment completion among CJI pregnant women discharged from outpatient treatment settings. Over 70% of discharges reported reduced substance use or maintained no use from treatment admission to discharge. Notably, less than one-third of discharges successfully completed treatment, which is consistent with recent research conducted among pregnant women with cannabis use disorder who utilized treatment services (30). Study results showed that discharges who were older were less likely to reduce their substance use during treatment. Furthermore, non-marijuana illicit primary substance use behaviors and comorbid mental illness were strongly and inversely related with treatment completion. A key finding of the present study was that self-help group attendance was associated with reduced substance use and successful treatment completion. The findings from the present study have notable implications for CJI pregnant women who utilize outpatient treatment services.

Regarding sociodemographic factors, our results demonstrated significant differences by age and race/ethnicity in reduced substance use. Women ages 25–34 and 35+ had lower odds of reduced substance use compared to women ages 18-24, potentially due to the connection between age, longer periods of substance use, and greater severity of use over time. Future research in this area should examine whether the observed relationship of age with treatment completion in the present study persists in other clinical samples.

Discharges who identified as Hispanic or as a race other than Black or White (classified as “Other”) were associated with higher odds of reduced substance use than discharges who were White. Given that the Other race/ethnicity category captured multiple races/ethnicities, the reasons for reduced substance use are not well understood. To date, there has been a dearth of research examining the impact of race and ethnicity among CJI pregnant women. A recent review of CJI women in outpatient substance use treatment only included women who were postpartum, highlighting the need for future research to examine racial/ethnic treatment outcomes and social determinants of health for CJI pregnant women (59). Prior research has found differences in recidivism based on race/ethnicity, with racial and ethnic minorities being more likely to be incarcerated and violate conditions of drug court and probation (60). The results of our study may indicate cultural differences of Hispanic pregnant women that positively affect treatment outcomes. Further research is needed to identify cultural factors that facilitate treatment motivation and positive treatment outcomes for demographic subpopulations.

Discharges who were unemployed had lower odds of treatment completion than discharges who were employed. Employed individuals may have had work-related requirements for treatment completion before returning, which may be a motivator for completing treatment and reducing substance use. This finding is supported by a previous study that demonstrated bidirectional benefits between employment and substance use treatment outcomes (61). As employment provides structure, accountability, and increases self-efficacy, research is needed to study the interaction of employment with treatment completion to determine if employment is a marker of one’s ability to adhere to treatment requirements, if increased financial security relieves socioeconomic stressors, or if other positive impacts of being employed makes treatment completion more likely. For example, Rumrill and Bishop (32) demonstrated that meaningful employment was associated with higher recovery outcomes and quality of life.

Several treatment-related factors were associated with reduced substance use and treatment completion. Specifically, self-help group attendance was found to be significantly associated with reduced substance use and treatment completion, which is supported across numerous studies (52, 62–65). Further research is needed to examine the mechanisms that underly the association between self-help group attendance and positive treatment outcomes. Such factors may include social support (63, 66), increased motivation for change, and alleviation of mental health-related issues. The data did not indicate whether self-help attendance was voluntary or mandated by the criminal justice system; nonetheless, this predictor was significantly associated with improved treatment outcomes. This study extends the literature supporting the relationship between self-help group attendance and improved outcomes for CJI pregnant women.

Peer support represents an opportunity for positive social contact that is linked with reduced substance use for those with CJI (67, 68). Some evidence suggests that incorporating peer support into treatment services has demonstrated effectiveness in promoting substance use behavior change for pregnant women (51, 69, 70). One self-help group model unique to pregnancy is to provide group antenatal/perinatal care alongside pregnancy and psychosocial education. Such approaches have demonstrated improved prenatal appointment attendance, increased trust and engagement with prenatal care, and increased patient satisfaction of the prenatal care experience among attendees (71–73). Studies examining group prenatal care among pregnant women with opioid use disorder have found increased positive health behaviors (e.g., breastfeeding) and bolstered ability to prevent relapse (73, 74). Integrating such peer recovery support models into formal treatment services may be a critical step to facilitating substance use recovery for CJI pregnant women.

This study found that primary marijuana use was associated with lower odds of reduced substance use compared to primary alcohol use, perhaps due to less perceived risk of marijuana use in pregnancy compared to other substances (75–77). Relatedly, primary opioid use and primary stimulant use were associated with lower odds of treatment completion compared to primary alcohol use. Among substance use treatment-mandated parolees, one study found that primary stimulant use was associated with a significantly lower likelihood of relapse (78). The reasons for differences in reduced substance use and treatment completion by primary substance use type are unclear, though it may be that the desire for behavior change or knowledge of alcohol’s adverse fetal effects may play contributing roles. Additionally, our findings may capture women using both opioids and stimulants, who represent a cohort demonstrated to be less likely to complete treatment, less likely to be able to access treatment, and more likely to overdose compared to use of either substance in isolation (79, 80).

Although detailing the reasons for treatment noncompletion was outside the scope of the study, incarceration represents an important endpoint of outpatient care. This is particularly relevant for CJI pregnant women, as criminal justice referrals are substantially more likely to be incarcerated during outpatient treatment (81). In addition, rates of recidivism vary across criminal justice referral sources. For example, persons in drug courts demonstrate lower rates of arrest compared to those in probation, jail, and prison (82). As such, research examining factors that influence incarceration during outpatient treatment episodes is needed. Additionally, the finding that there were regional differences in reduced substance use and treatment completion requires further study into underlying socio-ecological mechanisms. Specifically, future research should explore state-level differences in criminalization policies and geographic differences in the availability of quality treatment services.

### 4.1 Limitations

There are several limitations which merit consideration when interpreting the findings of the present study. TEDS-D data is cross-sectional, and the observed associations are not indicative of causation. The variables analyzed in this study should not be viewed as an exhaustive list of potential predictors of reduced substance use and treatment completion. This is because other variables not captured in the current study may also be related to these outcomes.

Since TEDS-D data is derived from state administrative databases, there may be heterogeneity across states in terms of data collection protocols and measurement definitions. TEDS-D data is sourced from publicly funded and state-licensed treatment settings, and study findings are not generalizable to private treatment centers or office-based settings which dispense medications for SUDs. Our results should also be interpreted within the context of outpatient treatment settings and not residential treatment or detox settings. Pregnancy status was only assessed at the time of admission in TEDS-D, and early or unrecognized pregnancies that are not captured at treatment admission may result in some women being mis-categorized as not being pregnant. Moreover, TEDS-D does not delineate information regarding pregnancy timing and treatment outcomes. For example, information on pregnancy trimesters at admission and whether birth occurred during treatment is not given. Study results only speak to outcomes for CJI women identified as being pregnant at treatment admission and do not account for the impact of specific pregnancy characteristics and pregnancy events on treatment outcomes.

Moreover, since the reduced substance use variable indicates if a person either maintained no use or reduced use of the substance primarily responsible for treatment from treatment admission to discharge, reduced use outcomes for secondary and tertiary substance use behaviors were not captured. It is also important to note that treatment discharges resulting in referral to other treatment services may constitute a positive outcome if the individual is linked to appropriate services, but it was not treated as such in the present study. While TEDS-D indicates if medication-assisted opioid therapy is included in the treatment plan and denotes the type of facility setting, it does not provide information on specific types of treatment modalities received during the treatment episode. Despite its relevance to treatment outcomes, polysubstance use was not assessed in study analyses due to the considerable number of substance use behaviors captured in TEDS-D data, which complicates categorizing specific polysubstance use combinations. Lastly, since one individual may account for multiple treatment discharges, the statistical assumption of independent observations was not met.

### 4.2 Conclusion

This study highlights sociodemographic and treatment-related factors that were identified as significant predictors of reduced substance use and treatment completion among CJI pregnant women discharged from outpatient treatment. In particular, discharges with self-help group attendance had significantly higher odds of reduced substance use and treatment completion compared to discharges who did not attend a self-help group. These findings indicate a clear value of self-help group attendance in fostering positive treatment outcomes, demonstrating a need for further research in this area. There are several program and policy implications of the study’s findings. One, there is a need for education among criminal justice and health care professionals about the factors that may contribute to improved treatment outcomes among CJI pregnant women with SUD. A complex web of needs exists among this population given the triple vulnerabilities of being CJI, pregnant, and having a SUD. Future research should consider examining factors of positive treatment outcomes that are relevant for specific types of substance use behaviors and/or SUDs. Longitudinal data sources with more robust measures of substance use behavior change are needed, particularly regarding substance use trajectories after treatment discharge. Lastly, the context of CJI matters, and considering how specific criminal justice system referral pathways relate to outpatient treatment outcomes is critical.

## Data Availability

The original contributions presented in the study are included in the article/supplementary material. Further inquiries can be directed to the corresponding author.
